# Mitochondrial Effects in the Liver of C57BL/6 Mice by Low Dose, High Energy, High Charge Irradiation

**DOI:** 10.3390/ijms222111806

**Published:** 2021-10-30

**Authors:** Brooke L. Barnette, Yongjia Yu, Robert L. Ullrich, Mark R. Emmett

**Affiliations:** 1 Department of Biochemistry and Molecular Biology, University of Texas Medical Branch (UTMB), Galveston, TX 77555, USA; brlawson@utmb.edu; 2 Department of Radiation Oncology, University of Texas Medical Branch (UTMB), Galveston, TX 77555, USA; yoyu@utmb.edu; 3 The Radiation Effects Research Foundation (RERF), Hiroshima 732-0815, Japan; ullrich@rerf.or.jp; 4 Department of Pathology, University of Texas Medical Branch (UTMB), Galveston, TX 77555, USA; 5 Department of Pharmacology and Toxicology, University of Texas Medical Branch (UTMB), Galveston, TX 77555, USA

**Keywords:** space radiation, liver, systems biology, integrated omics, mitochondrial dysfunction

## Abstract

Galactic cosmic rays are primarily composed of protons (85%), helium (14%), and high charge/high energy ions (HZEs) such as ^56^Fe, ^28^Si, and ^16^O. HZE exposure is a major risk factor for astronauts during deep-space travel due to the possibility of HZE-induced cancer. A systems biology integrated omics approach encompassing transcriptomics, proteomics, lipidomics, and functional biochemical assays was used to identify microenvironmental changes induced by HZE exposure. C57BL/6 mice were placed into six treatment groups and received the following irradiation treatments: 600 MeV/n ^56^Fe (0.2 Gy), 1 GeV/n ^16^O (0.2 Gy), 350 MeV/n ^28^Si (0.2 Gy), ^137^Cs (1.0 Gy) gamma rays, ^137^Cs (3.0 Gy) gamma rays, and sham irradiation. Left liver lobes were collected at 30, 60, 120, 270, and 360 days post-irradiation. Analysis of transcriptomic and proteomic data utilizing ingenuity pathway analysis identified multiple pathways involved in mitochondrial function that were altered after HZE irradiation. Lipids also exhibited changes that were linked to mitochondrial function. Molecular assays for mitochondrial Complex I activity showed significant decreases in activity after HZE exposure. HZE-induced mitochondrial dysfunction suggests an increased risk for deep space travel. Microenvironmental and pathway analysis as performed in this research identified possible targets for countermeasures to mitigate risk.

## 1. Introduction

In 1948, Von Braun wrote the nonfiction scientific book, *The Mars Project*, about a manned mission to Mars which sparked fascination in traveling deeper into our galaxy. It is now hoped that this mission will be possible by the year 2030; however, with that hope, first, there are several issues that must be addressed. One of the most eminent risks is exposure to galactic cosmic rays (GCRs) which contain low levels (<1%) of high charge/high energy ions (HZEs) which can be a tremendous health risk due to the possibility of carcinogenesis. Unlike low-linear energy transfer (LET) radiation such as gamma rays and X-rays, HZEs have much more densely ionizing radiation, and therefore are more damaging to tissues and cells. Although a GCR is comprised of only ~1% HZEs, these ions possess significantly higher ionizing power with greater potential for radiation-induced damage.

Reactive oxygen species (ROS) have been suggested to be generated secondarily following exposure to ionizing radiation from biological sources such as mitochondria. ROS have a variety of biological roles including apoptotic signaling [1], genomic instability [2], and radiation-induced bystander effects that ultimately impact cellular integrity and survival. It is unclear exactly how the mitochondria are responsible, but it is thought that it is due to leakage of electrons from the electron transport chain that results in the generation of superoxide radicals (O_2_^−^) through their interaction with molecular oxygen [3,4]. Mitochondria, similar to most other biological systems, do not operate at 100% efficiency. Thus, electrons are occasionally lost, and ROS are produced. ROS produced from mitochondria are a normal occurrence. In fact, mitochondria are the largest source of ROS in the cell, but they also have the machinery to be the best ROS scavengers in the cell. Problems arise when the mitochondria are damaged and the electron leakage results in more ROS than can be scavenged. In 2012 and 2013, Datta et al. [5,6] studied 2 Gy and 5 Gy gamma irradiation and 1.6 Gy and 4 Gy ^56^Fe irradiation in mice. Their results showed that radiation quality affected the level of persistent oxidative stress with higher elevations of intracellular reactive oxygen species (ROS) and mitochondrial superoxide in ^56^Fe-irradiated as compared with non-irradiated and gamma-irradiated groups. Additionally, NADPH oxidase activity, mitochondrial membrane damage, and loss of membrane potential were greater in ^56^Fe-irradiated mice livers.

In this study, a data-rich systems biological approach incorporating transcriptomics (deep RNA sequencing), proteomics, lipidomics, and functional bioassays was used to investigate the microenvironmental changes in the livers of C57BL/6 mice induced by low dose HZE irradiation (600 MeV/n ^56^Fe (0.2 Gy), 1 GeV/n ^16^O (0.2 Gy), or 350 MeV/n ^28^Si (0.2 Gy)). The results showed alterations in mitochondrial function in all levels of the interactive omics datasets, demonstrating that low dose HZE exposure, similar to doses that could be accumulated during a long duration deep space mission, induces significant mitochondrial dysfunction.

## 2. Results

**Figure 1:**
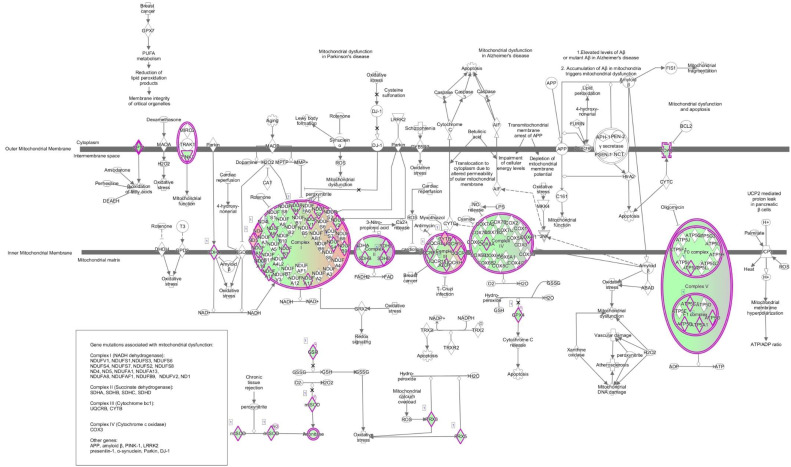
Data collected from transcriptomic and proteomic experiments were imported into IPA. One of the most prominent pathways seen in one-month ^16^O-irradiated C57BL/6 mice livers in the transcriptomic data was mitochondrial dysfunction. 46 transcripts in this pathway were dysregulated as compared with the non-irradiated control. Green indicates down regulation and red indicates up regulation. The brighter the color, the more extreme the altered regulation.

The data collected from transcriptomic and proteomic experiments were imported into the ingenuity pathway analysis (IPA). Several pathways involved in mitochondrial function were found to be altered after HZE irradiation including the mitochondrial dysfunction pathway. As shown in Figure 1 , mitochondrial dysfunction was one of the most prominent pathways with 46 transcripts being dysregulated in the transcriptomic data of one-month ^16^O-irradiated mice livers.

Table 1 shows the transcripts and proteins that were dysregulated within the mitochondrial dysfunction pathway for each irradiation treatment and timepoint. HZE exposure also affected other significant pathways. Table 2 shows the top five affected canonical pathways and the top five upstream regulators along with some other important pathways in the transcriptomic and proteomic datasets. Several of the affected pathways found both in the transcriptomic and proteomic datasets have links to mitochondrial function. Mitochondrial stress accompanies ROS production and ATP decline, as well as an accumulation of unfolded protein, decrease in Ca2+ buffering, alteration of metabolites in the TCA cycle, oxidative phosphorylation, fatty acid oxidation, etc. [7]. As seen in Table 2, the transcriptomic data show many pathways within the early timepoints that are linked to mitochondria. These pathways include sirtuin signaling, ER stress, unfolded protein response, L-carnitine shuttle, TCA cycle, ubiquinol-10 biosynthesis, acute phase response, EIF2 signaling, NRF2-mediated oxidative stress response, and amino acid metabolism (e.g., asparagine biosynthesis). The FXR/RXR and LXR/RXR pathways are also affected. Although some of these pathways also changed in the gamma-irradiated mice, they mostly changed in the later post-irradiation time points, similar to changes noted in the gamma-irradiated mitochondrial dysfunction assays which monitored Complex I activity (discussed below).

Within the proteomic datasets, there are correlations with the transcriptomic results such as sirtuin signaling, acute phase response, L-carnitine shuttle, unfolded protein response, and amino acid biosynthesis (e.g., L-glutamine biosynthesis). In addition, the proteomic data show changes in calcium transport which is essential for both ER and mitochondrial function. Additionally, of note are changes in immune-related pathways such as NFκB and JAK family kinases in IL-6 type cytokine signaling. When the ROS level is elevated, it can activate NF-κB which ultimately produces proinflammatory cytokines such as interleukin-6 (IL-6) [8]. The FXR/RXR and LXR/RXR pathways are also seen within both datasets. Unique to the proteomic data are leukocyte extravasation signaling, glycogen degradation, endocytosis signaling, ILK signaling, and phagosome maturation.

**Figure 2:**
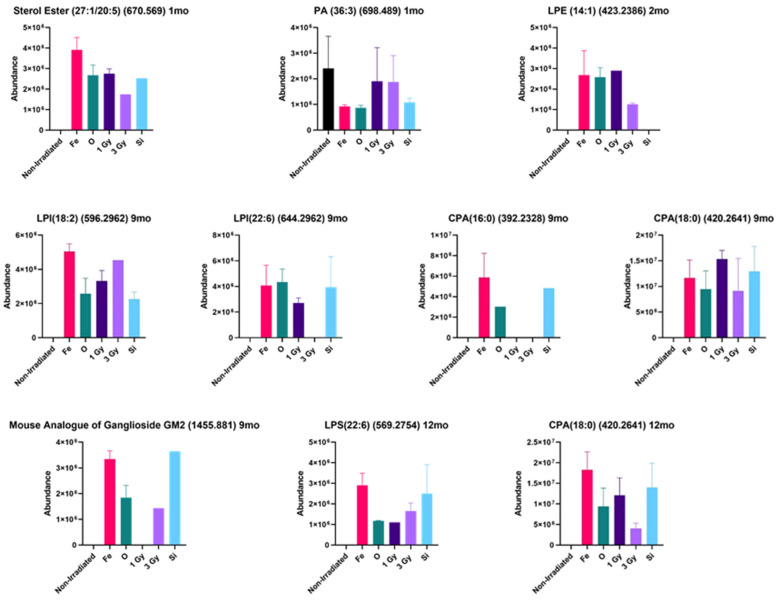
HZE-induced changes in lipid species throughout the time course. Figure 2 shows a representative selection of some of the identified lipids that are biologically linked to mitochondrial function that had modified expression as compared with the non-irradiated control. In many cases, the lipids of interest were not detected in the non-irradiated control, but were highly induced in the livers of the irradiated mice. All data were from a group of 5 mice.

The lipidomic data also supported the mitochondrial dysfunction and increased immune response after irradiation, as was found in the transcriptomic and proteomic results. Figure 2 shows data for liver lipids that exhibited substantial changes after irradiation throughout the time course. It is important to note that a significance value for most of the increased changes could not be assigned to the lipid increases since many of the lipid species were not detected in the non-irradiated mice, and thus a statistical analysis could not be performed. As compared with non-irradiated control, the increases in many lipids were quite large after HZE irradiation.

At 1 month post-irradiation, an increase in sterol ester (27:1/20:5) was highest in the ^56^Fe-irradiated mice livers, and phosphatidic acid (PA) (36:3) was decreased primarily in the ^56^Fe- and ^16^O-irradiated mice livers as compared with the non-irradiated control. At 2 months, lysophosphatidylethanolamine (LPE) (14:1) was increased in the irradiated mouse livers. It should be noted that LPE was detected in only one of the five mouse liver samples in the group (*n* = 5) in 1 Gy liver samples, thus, if the data points were removed, the LPE levels would be the highest in ^56^Fe- and ^16^O-irradiated mouse livers. At 9 months post-irradiation, cyclic phosphatidic acid (CPA) (16:0), CPA (18:0), lysophosphatidylinositol (LPI) (18:2), LPI (22:6), and GalNAcβ1-4(NeuGcα2-3) Galβ1-4Glcβ-Cer (d18:1/22:0) were all increased relative to the non-irradiated control. CPA (16:0) was only detected in the HZE-irradiated samples in the 9 months post-irradiated mouse livers. The increase in GalNAcβ1-4(NeuGcα2-3) Galβ1-4Glcβ-Cer (d18:1/22:0) was of particular interest because this glycosphingolipid is the mouse analogue of the human ganglioside GM2 (ganglioside GM2) (t18:0/22:1) (13Z) and was detected in the mouse livers of animals irradiated with ^56^Fe, ^16^O, and ^28^Si. This glycosphingolipid was only detected in one of the five mouse liver samples in the group (*n* = 5) in 3 Gy gamma-irradiated samples. At 12 month post-irradiation, CPA (18:0) and lysophosphatidylserine (LPS) (22:6) were both increased relative to the control. CPA (18:0) was only increased in irradiated mouse livers and was highest in ^56^Fe-irradiated group. LPS (22:6) was only increased in the irradiated samples and was highest in the ^56^Fe-irradiated group and only one of the five mouse liver samples in the (*n* = 5) ^28^Si-irradiated group.

**Figure 3:**
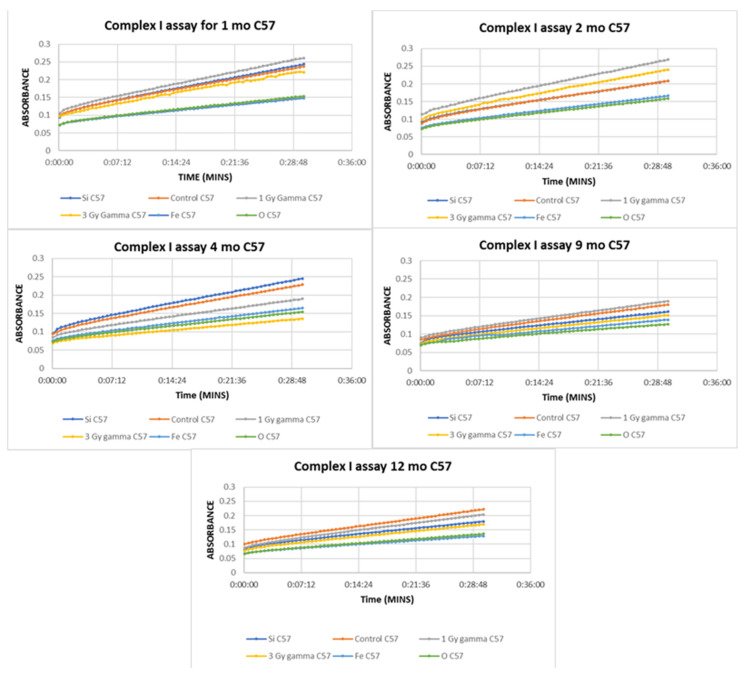
Mitochondrial Complex I activity of C57BL/6 mice at 1 month post-irradiation exhibited a decrease in ^16^O- and ^56^Fe-irradiated mice livers as compared with the non-irradiated control. All slopes are significantly different (*p* < 0.0001) except for ^28^Si and non-irradiated (*p* = 0.5600) as well as ^56^Fe and ^16^O (*p* = 0.3964). At 2 months post-irradiation, similar decreases in ^16^O- and ^56^Fe-irradiated mice livers were observed as compared with the non-irradiated control. All slopes are significantly different (*p* = 0.0005 for ^56^Fe and ^16^O and *p* < 0.0001 for all others) except for ^28^Si- and non-irradiated (*p* = 0.9981) mice livers. At 4 months post-irradiation, a decrease was seen in ^16^O, ^56^Fe, 1 Gy gamma, and 3 Gy gamma as compared with the non-irradiated control. All slopes are significantly different (*p* < 0.0001). At 9 months post irradiation, a decrease was seen in all irradiation types as compared with the non-irradiated control except for 1 Gy gamma. All slopes are significantly different (*p* < 0.0001). At 12 months post-irradiation, a decrease was seen in all irradiation types as compared with the non-irradiated control. All slopes are significantly different (*p* = 0.0158 for ^56^Fe and ^16^O and *p* < 0.0001 for all others). All data points represent a group of *n* = 3 and correlate to the same animals from which the transcriptomic and proteomic data were generated.

To validate the mitochondrial dysfunction reflected in the above integrated omics datasets, functional mitochondrial assays for Complex I of the electron transport chain were performed on the same liver tissues; Complex I catalyzes the first step in the electron transport chain. An enzyme oxidizes NADH transferring an electron to ubiquinone which is an electron carrier embedded in the lipid bilayer of the inner mitochondrial membrane. In the Complex I assay, capture antibodies specific for Complex I coat the wells of the plate so that Complex I is selected from the mitochondrial extract. The assay works by measuring the oxidation of NADH to NAD+ with simultaneous reduction of the provided dye. Thus, the more NAD+ that is produced, the more yellow the dye will become resulting in an increase in absorbance. The results from this assay (Figure 3 ) indicate a decrease in activity of Complex I in both the ^56^Fe- and ^16^O-irradiated samples as compared with the non-irradiated control throughout the time course. Complex 1 activity was not altered in 1 Gy and 3 Gy gamma-irradiated mice until the four-month timepoint. At 9 months, there was no longer a decrease in function of the 1 Gy gamma, but the decrease returned at 12 months. ^28^Si also showed a decrease at 9 months and it continued through the final timepoint. Previous studies have shown significant decreases in Complex I activity and it has been suggested this Complex may be involved in the initiation of mitochondrial biogenesis, and thus a decrease in Complex I activity would lead to decreased mitochondrial biogenesis. Dysfunction of this particular complex is the main cause of several mitochondrial diseases and disorders [4].

Mitochondrial dysfunction has been known to include a decrease in mitochondrial DNA copy numbers as well as reduced mRNA concentration of genes encoding mitochondrial proteins and decreased antioxidant capacity [9]. To investigate this, mitochondrial copy numbers were measured via qt-PCR in all samples. While there were trends in the data that showed slight decreases of mitochondrial DNA in ^56^Fe, ^16^O, and 1 Gy gamma at 1 month post-irradiation, the data were not statistically significant from the non-irradiated control (data not shown). The decreases likely did not reach significance due to individual variability. To fully determine if the copy numbers were being affected, this experiment would require a greater number of mice.

Cardiolipins are known to be primarily found in the inner mitochondria membrane and are required for mitochondrial function. They are involved in maintaining membrane potential and architecture and are also known to provide essential structure and functional support to several proteins that are involved in mitochondrial bioenergetics. Loss of cardiolipin content, alterations in its acyl chain composition, and or cardiolipin peroxidation have been previously associated with mitochondrial dysfunction [10]. Changes in cardiolipins will alter membrane fluidity and the folding of the inner mitochondrial membrane, and can alter the organization and function of the respiratory complexes. These molecules help organize the respiratory complexes into super complexes to facilitate optimal electron transfer among redox partners. Many of the complexes and carrier proteins require cardiolipins for proper assembly and function. Loss of these lipids and their peroxidation have been associated with both aging and several metabolic and degenerative diseases [11]. Since our lipidomic platform was focused on global lipid levels in the whole liver instead of being focused on mitochondrial specific lipids, we utilized a fluorescence cardiolipin assay to obtain information on this vital class of lipids in isolated mitochondria. Slight decreases (results not shown) in cardiolipin levels were seen at one-month post HZE irradiation, at 9 months for ^56^Fe and ^16^O irradiation, and in all radiation types at 12 months post-irradiation, but none of these changes were statistically significant. The lack of statistical significance could be due to the small number as was proposed for the lack of significance for the decrease in mitochondrial copy numbers. It is also important to note that the cardiolipin assay used in these studies detects both normal cardiolipins and oxidized cardiolipins. Thus, total cardiolipin levels measured with this assay does not distinguish oxidation state of the cardiolipins.

## 3. Materials and Methods

The chemicals used in this study were of the highest possible purity and all solvents were LC-MS grade or better. Most high purity chemicals were ordered from Sigma-Aldrich (St. Louis, MO, USA), unless otherwise stated in the subsequent Methods sections.

**Figure 4:**
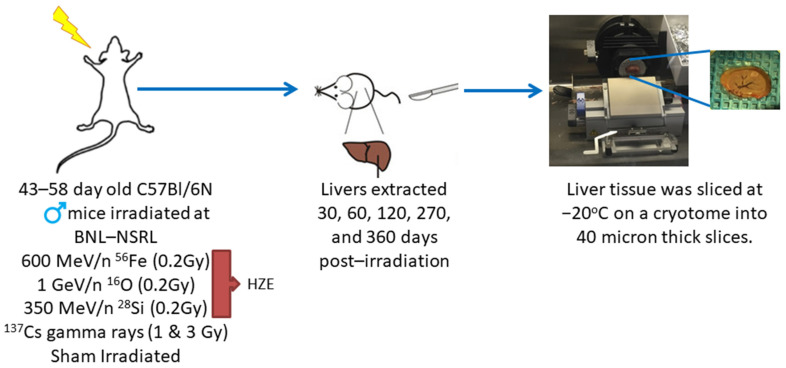
C57Bl/6N mice were placed into 6 treatment groups and received the following irradiation treatments at BNL-NSRL: 600 MeV/n ^56^Fe (0.2 Gy), ^137^Cs (1.0 Gy) gamma rays, ^137^Cs (3.0 Gy) gamma rays, 1 GeV/n ^16^O (0.2 Gy), 350 MeV/n ^28^Si (0.2 Gy), and sham irradiation. Liver tissues were collected at 30, 60, 120, 270, and 360 days post-irradiation, rapidly frozen at −78.5 °C, and sliced on a cryotome for experimental platforms.

For the animal model and irradiations, C57BL/6 mice (43–58 days old) were purchased from Charles Rivers (Wilmington, MA) and were shipped directly to Brookhaven National Laboratory (BNL). All studies had prior approval from both the UTMB and the BNL Institutional Animal Care and Use Committee (IACUC). Irradiations were performed at the NASA Space Radiation Laboratory (NSRL), as previously described in [12]. After irradiation, the mice were shipped to Galveston, Texas where they were housed in the Animal Care Facilities at the University of Texas Medical Branch (UTMB) until they were euthanized. Twenty-five C57BL/6 male mice were placed in each of the 6 groups and received the defined irradiation treatment. The 6 treatment groups consisted of: 600 MeV/n ^56^Fe (0.2 Gy), 1 Ge V/n ^16^O (0.2 Gy), 350 MeV/n ^28^Si (0.2 Gy), ^137^Cs (1.0 Gy) gamma rays, ^137^Cs (3.0 Gy) gamma rays, and sham irradiation. The radiation doses were selected based on previous work by Weil et al. [13] and through direct discussions with NASA. As shown in Figure 4 mice were euthanized, and livers were extracted at 30, 60, 120, 270, and 360 days post-irradiation. Tissues were rapidly frozen on aluminum blocks held at dry ice temperature (−78.5 °C), and then stored at −80 °C until the samples could be processed. Two 40-micron slices were taken on a cryotome at −20 °C for each experimental platform. Cryotome slicing of the liver samples permitted multiple samples to be taken from each liver without ever going through a freeze/thaw cycle, thus, preserving sample integrity.

For the proteomic studies, tissue slices were lysed with RIPA buffer mixed with Halt protease inhibitor EDTA-free, Halt phosphatase inhibitor cocktail, and Pierce universal nuclease [14] (Thermo Fisher, Waltham, MA, USA) and homogenized on ice with a polytron equipped with a microgenerator (20 s × 1, @ 10,000 rpm). Samples were incubated on ice for 30 min and briefly vortexed twice during incubation, and then centrifuged at 15,000× *g* for 20 min at 4 °C. Protein concentration of the supernatant was determined with a Pierce BCA Protein Assay Kit (Thermo Scientific, Rockford, IL, USA). A volume of supernatant that contained 100 ug of protein was removed, reduced, and alkylated. Ten microliters of 200 mM tris (2-carboxyethyl) phosphine (TCEP) diluted with 50 mM triethylammonium bicarbonate (TEAB) was added to each sample and incubated at 55 °C for 1 h while mixing. Ten microliters of 375 mM iodoacetamide was added and incubated in the dark at room temperature for 45 min while mixing. Proteins were precipitated overnight at −20 °C with 880 µL of ice-cold acetone. The samples were centrifuged at 15,000× *g* for 20 min at 4 °C. The supernatant was decanted, and samples were de-lipidated by adding 1 mL of ice-cold (tri-n-butylphosphate/acetone/methanol, 1:12:1) [15] and incubated for 90 min on ice. The samples were centrifuged at 2800× *g* for 15 min at 4 °C. The supernatant was removed and 1 mL of ice-cold tri-n-butylphosphate was added. The samples were centrifuged again under the same conditions as previously stated. The supernatant was removed and 1 mL of ice-cold acetone was added. Centrifugation was repeated and the supernatant removed. One milliliter of ice-cold methanol was added and the samples were centrifuged for a final time. The sample pellets were air-dried and resuspended in 12.5 µL of 8 M urea. Four mg of trypsin in 50 mM TEAB was added to each sample and incubated for 24 h at 37 °C. The samples were desalted using C18 Sep-Pak Vac 1cc cartridges attached to a vacuum manifold. The cartridges were equilibrated using three 1 mL aliquots of acetonitrile at a flow rate of ~2 mL/min. The cartridges were washed/equilibrated with three 1 mL aliquots of 0.25% trifluoroacetic acid. Trifluoroacetic acid was added to the samples to bring them to a final concentration of 1%. The samples were loaded on to Sep-Pak cartridges and allowed to pass via gravity flow. The cartridges were washed with four 1 mL aliquots of 0.25% trifluoroacetic acid. The peptides were eluted in 1 mL of 80% acetonitrile/0.1% formic acid by gravity flow and dried in a SpeedVac Concentrator.

For the spectral library generation, 40 µg of protein was taken from each of the proteomic samples and mixed together. Then, the 400 µg aliquot of the mixture was taken for fractionation on an Agilent Technologies 1260 Infinity HPLC. The sample aliquot was lyophilized to dryness and reconstituted in 125 µL of 85% acetonitrile/56 mM formic acid (aqueous) which was fractionated over a hydrophilic interaction chromatography (HILIC) column (PolyLC PolyHYDROXYETHYL A, PolyLC Inc., Columbia, MD, USA), 200 × 4.6 mm, particle size 5 µm. Buffer A was 85% acetonitrile/56 mM formic acid, pH 3.0 and Buffer B was 8.5 mM ammonium formate/56 mM formic acid, pH 3.0. Fifty-three 1 mL fractions were collected at a flow rate of 1 mL/min under the following gradient conditions: column equilibrated with ten column volumes of 100% A, 400 µg sample was loaded and washed with four column volumes of 100% A, sample collection began with 3.5 column volumes to 15% B, four column volumes to 40% B, and finished with 10 column volumes to 100% B. The fractions were all lyophilized to dryness and Fractions 21–40 were reconstituted in 25 µL of 98% H_2_O, 2% acetonitrile, and 0.1% formic acid. These fractions were pooled with corresponding Fractions 1–20. For example, Fractions 1 and 40, 2 and 39, 3 and 38, etc. Fractions 41–53 were taken up into 12.5 µL, individually. Then, all samples, individual or pooled, were quantitated (2 µL) by BCA on a Thermo Nanodrop for peptide concentration. Fraction pairs 4 and 37, 5 and 36, 6 and 35, 7 and 34, and 8 and 33 were spun down at 12,000 g for 5 min. Eighteen µL of the remaining 23 µL was added to autosampler vials and 6 µL was injected onto the column. The samples were analyzed by data-dependent acquisition (DDA) mass spectrometry (MS). The other fractions were further pooled to achieve the desired peptide concentration that was at least 0.1 mg/mL or greater. The pooled groups were as follows: Fraction 1, 40, 19, 22, 9, and 32; 20, 21, 17, 24, 15, and 26; 14, 27, 10, 31, 2, 39, 12, and 29; 16, 25, 18, 23, 13, 28, 11, and 30; and finally, 41–53. These combined fractions were analyzed by DDA MS. The data from all 10 fractions were combined to search mouse UKB DB. Then, these files were used to create the spectral/ion library.

For the proteomic analysis, a chromatographic separation and mass spectrometric analysis was performed with a nano-LC chromatography system (Thermo Dionex Ultimate 3000 RSLC nano system, Thermo Fisher, Waltham, MA, USA) interfaced to an AB Sciex Triple Time-of-Flight (TOF) 5600 mass spectrometer. The samples were analyzed by LC-MS/MS at a flow rate of 300 nL/min. The samples were separated over an Acclaim PepMap 100 C18 nano-LC column, 75 microns ID and 250 mm in length (Thermo Fisher, Waltham, MA, USA). Then, 1 µg of protein from each sample was injected onto the column. The gradient started at 97%/3% A/B ramping to 20%/80% A/B over 72 min; 20%/80% A/B was held for 6 min, and then re-equilibrated to 97%/3% A/B, and held for 25 min. Solvent compositions were: Solvent A, 100% H_2_O with 0.1% formic acid and Solvent B, 100% acetonitrile with 0.1% formic acid. The gradient profile was completed in 105 min. A custom isolation scheme was used over the mass range of 400–1200 *m*/*z* so that smaller isolation windows could be applied in mass ranges that were known to have the highest concentration of peptides. A rolling collision energy was used for MS/MS acquisition. The samples were run in block randomized order. The ion library was imported in PeakView (Sciex) followed by individual samples for all conditions. Retention time (RT) alignment process settings were as follows:

**Peptide Filter**

Number of peptides per protein, 15;
Number of transitions per peptide, 5;
Peptide confidence threshold %, 95;
False discovery rate threshold %, 1.0.

**XIC Options**

XIC extraction window (min), 8.0;
XIC width (ppm), 30.

The RT standards were selected from spiked in Pep Cal Mix (PCM) and carbamoyl-phosphate every 5–10 min during the duration of the run for RT calibration. Once selected, the RT fit was calculated, and points were deleted and added as necessary so that the best fit was achieved. After the RT calibration was complete, processing was continued. Then, peak areas were exported to MarkerView (Sciex) where a statistical analysis by pairwise comparisons was performed between control and treated groups. The proteomic analysis identified ~3200 proteins per sample. Lists were imported into IPA and the filtering parameter was set at a fold change of ≥1.15.

For RNA sequencing, the total RNA was isolated from two 40-micron liver slices via phenol-free kits using an RNAqueous kit (Invitrogen, Vilnius, Lithuania). RNA was monitored for yield and quality via a Nanodrop spectrophotometer (Thermo Scientific, Waltham, MA, USA) and an RNA 1000 chip on an Agilent 2100 Bioanalyzer (Agilent Technologies, Santa Clara, CA, USA). rRNA was removed via Ribo-Zero Gold rRNA removal kits (Human/Mouse/Rat) from Illumina.

To create the cDNA libraries, mRNA from samples were selected from total RNA (0.5–2.0 µg) using poly dT primers that recognize the polyA tail. mRNA was fragmented using divalent cations and heat (94 °C, 8 min). Illumina TruSeq V2 sample preparation kits were used for library construction. Fragmented PolyA+ samples were converted to cDNA by random primed synthesis using superscript II reverse transcriptase (Invitrogen). Following second strand synthesis, the double strand DNAs were treated with T4DNA polymerase, 5′ phosphorylated, and an adenine residue was added to the 3′ ends. Then, adapters were ligated to the ends of the target template DNAs. After ligation, the template DNAs were amplified using primers specific to each of the non-complimentary sequences in the adapter. This creates a library of DNA templates that have non-homologous 5′ and 3′ ends. Fifty base pair reads were acquired on the Illumina HiSeq 1500 and fed into the NEB RNA Ultra Library Kit for Illumina to complete the library. The samples were clustered onto the flow cell using the cBot and sequenced on the HiSeq-1500 as a paired-end run with 50 × 50 bp lengths in high output mode. Reads were aligned with the STAR alignment program using the ENCODE recommended parameters. Reads per gene were counted using the –quantMode GeneCounts option. PIVOT version 1.0.0 (Junhyong Kim Lab, University of Pennsylvania) was used for differential expression analysis. Within PIVOT, RLE(DeSeq) was used for data normalization and an exact test with false discovery rate (FDR) set to 0.1 was used to compare control groups to treatment groups via experiment design/condition. The RNAseq data quantified ~51,000 mRNA transcripts per sample. Then, the acquired lists were imported into IPA.

For the lipidomic studies, two 40-micron mouse liver tissue slices were homogenized in 400 µL of 155 mM ammonium acetate [16] solution on ice using a Polytron equipped with a microgenerator (10 s × 2, @ 15,000 rpm). A 2 µL volume was removed from the homogenate and diluted in 155 mM ammonium acetate (typically 2 µL of sample in a total volume of 4.5 µL) for BCA total protein determination. For BCA, 2 µL of diluted sample was combined with 20 µL of working reagent and read on a Thermo Nanodrop. A volume corresponding to 200 µg of total protein was transferred to a 2 mL screw cap (Teflon lined) glass vial and 1:1 MeOH/CHCl_3_ (400 µL of each solvent) was added. The MeOH solution contained 2 mM butylated hydroxytoluene (BHT) to prevent lipid oxidation [17]. The samples were placed in a sonicating water bath for 30 min, and then transferred to a shaking heat block at 48 °C where they remained overnight. After removal from the heating block, the samples were placed in a sonicating water bath for 10 min. The samples were centrifuged at 5000× *g* for 15 min at room temperature. The supernatant was transferred to a 30 mL glass Corex tube, capped with a piece of aluminum foil and saved for later (can be stored at room temperature). Then, 1:1 MeOH/CHCl_3_ (400 µL of each solvent) was added to the pellet in the vial, and the 10 min sonication step and 15 min centrifugation step were repeated. The supernatant was combined with the previous aliquot in the 30 mL Corex tube. Then, 1:1 MeOH/CHCl_3_ was added to the pellet once more and the process was repeated. To the combined supernatant in the Corex tube, 3.3 mL of H_2_O and 1.2 mL of CHCl_3_ were added. The mixture was vortexed and mixed well with the aid of a glass pipet. The Corex tube with combined aliquot was centrifuged at 5000× *g* for 20 min at room temperature to produce 2 phases with clear separation. Polar lipids were in the aqueous layer (top layer). This layer was transferred to 2 mL screw cap glass vials and dried in a SpeedVac Concentrator. The lower (non-polar) layer was transferred to a 4 mL screw cap (Teflon-lined) glass vial and stored a −80 °C for future use. The dried polar layer was reconstituted in 100 µL of 80% MeOH, 20% H_2_O with 10 mM NH_4_OAc for analysis by ultra-high resolution mass spectrometry [18]. Chromatographic separation and mass spectrometric analyses were performed with a nano-LC chromatography system (Eksigent nanoLC 2D system) interfaced to a 12T Bruker Solarix Fourier Transform ion cyclotron resonance (FT-ICR MS) mass spectrometer. The samples were analyzed by nanoLC-MS/MS at a flow rate of 400 nL/min. The samples were separated over an in-house packed, 75 micron ID, nano-LC column packed with 8 cm of phenyl hexyl resin (Phenomenex, Torrence, CA, USA). Five microliters of each sample was loaded onto the column and washed for 5 min with 20%/80% A/B solvent. The sample was eluted with a gradient starting at 20%/80% A/B solvent and ramping to 1%/99% A/B solvent over 10 min; 1%/99% A/B solvent was held for 5 min to elute everything off the column. Then, the solvent was stepped down immediately to 20%/80% A/B solvent, and held there for 10 min to re-equilibrate the column for the next sample. The total gradient profile (load/sample, wash/gradient, elute/column, wash/column, re-equilibrate) lasted for a total of 30 min. The solvent compositions were: Solvent A, 98% H_2_O, 2% MeOH, with 10 mM NH_4_OAc and Solvent B, 98% MeOH, 2% H_2_O, with 10 mM NH_4_OAc) [13]. MS/MS was conducted at 20V collision energy. The samples were all run in block randomized order. The data were processed via Bruker’s Data Analysis 4.0. The SNAP algorithm was implemented for peak picking and charge state determination. Lipid identification was conducted by searching neutral state masses in the LIPIDMAPS structural database (LMSD) as well as the computationally generated database of ”bulk” lipid species (COMP_DB) [19]. The lipid analysis identified ~800 lipids per sample. Then, the lipids of interest were targeted for statistical analysis using a t-test to compare the respective non-irradiated control to each irradiated condition using PRISM 8 version 8.4.2.

For the mitochondria studies, mitochondria were isolated from four 40-micron liver slices via mitochondrial isolation kits (Abcam, Cambridge, UK). Protease inhibitor was added to isolation buffer (1:100). One milliliter of isolation buffer was added to each sample and homogenized on ice using a Polytron equipped with a microgenerator (10 s × 1, @ 15,000 rpm). The homogenates were transferred to a 2 mL centrifuge tube and spun at 1000 *g* for 10 min at 4 °C. The supernatant was transferred to a fresh tube and spun at 12,000 *g* for 15 min at 4 °C. The supernatant was decanted, and pellet was washed and resuspended in 500 µL of isolation buffer. The samples were again spun at 12,000 *g* for 15 min at 4 °C and the previous step was repeated. Once the pellet was resuspended in 500 µL of isolation buffer, the process was repeated once more. The final pellet was resuspended in 200 µL of isolation buffer and BCA was used to determine protein concentration.

For the Complex I assay, an Abcam Complex I Enzyme Activity Microplate Assay Kit (Colorimetric) was used to measure mitochondrial Complex I activity. Isolated mitochondrial samples were diluted with isolation buffer, to final concentrations of 400 µg/µL and 200 µL, were loaded on the assay plates. The plates were incubated for 3 h at room temperature, and then were washed with 300 µL of 1X buffer, three times. Then, 200 µL of assay solution was added to each well and optical density was measured on a Synergy H4 Hybrid Multi-Mode Microplate Reader (BioTek, Winooski, VT) in kinetic mode for 30 min with a reading taken every 30 s. Using Microsoft excel, replicates were averaged and plotted using the function, scatter with straight lines and markers. Slopes were compared using the analysis of covariance in R Studio version 1.1.456. Since the results indicated that all the slopes were different, the emmeans package was, then, used to determine where the differences lie.

For the RTqPCR analysis of mitochondrial DNA, DNA was isolated from small liver samples (approximately the size of a grain of rice) with DNeasy Blood and Tissue Kits from Qiagen (Germany). One hundred and eighty microliters of Buffer ATL and 20 µL of proteinase K were added and the samples were incubated overnight at 56 °C to complete tissue lysis. The following day, isolation was completed following the kit protocol. Then, the samples were analyzed on a Thermo Nanodrop spectrophotometer to determine concentration and purity. The samples were ultimately diluted to a final concentration of 0.1 ng/µL. The primers used were:

**The Mt CO1 primers**

Forward: 5-TGC TAG CCG CAG GCA TTA C-3;
Reverse: 5-GGG TGC CCA AAG AAT CAG AAC-3.

**The NDUFV1primers**

Forward: 5-CTTCCCCACTGGCCTCAA G-3;
Reverse: 5-CCA AAA CCC AGT GAT CCA GC-3 [20].

A master mix of each primer was made for each plate using 250 µL of H_2_O, 100 µL of primer, and 500 µL of iTaq Universal SYBR Green Supermix (BioRad, Hercules, CA). The samples were run in triplicate. Then, 51 µL of master mix and 9 µL of DNA were placed in the first well and thoroughly mixed, and then 20 µL of the solution was transferred into a second and third well. This was repeated for each sample with both sets of primers. The PCR cycle was as follows: 94 °C × 10 min to initiate and 40 cycles of 94 °C × 10 sec and 60 °C × 30 s [21]. The analysis was performed on a CFX96 Real-Time System (BioRad) with a C1000 Touch Thermal Cycler. Replicates for each primer were averaged and the ΔCt was calculated, which is equal to the counts via the nuclear primer minus the counts from the mitochondrial specific primer. The ratio mtDNA/nDNA was calculated using the formula 2 × 2^ΔCt^. The calculated values were graphed in Prism 6.07 and were analyzed via one-way ANOVA at each timepoint. The ratio values determined by PCR were also grouped with their corresponding values from the complex assay (slope from Complex I assay/PCR ratio). These values were also graphed in Prism 6.07 and were analyzed via one-way ANOVA at each timepoint.

For the cardiolipin assay, Cardiolipin Assay Kits (Fluorometric) (BioVision, Milipitas, CA) were used to determine the amount of cardiolipin present in the liver mitochondrial samples. A volume corresponding to 5 µg of protein from a mitochondrial sample previously isolated from mice liver was loaded into a well on the microtiter plate to be used as the “sample” and another aliquot containing the same amount was used as the “sample background control”. The “sample” wells were brought up to a final volume of 50 µL using the reaction mix which contained 2:50 cardiolipin probe to cardiolipin buffer. The “sample background control” wells were brought up to a final volume of 100 µL using the cardiolipin buffer. The plates were incubated for 10 min, and the optical density was measured on a Synergy H4 Hybrid Multi-Mode Microplate Reader (BioTek), Ex/Em 340/480 nm. The “sample background control” was not higher than the 0 mM reading for any of the samples, therefore, only the 0 mM reading was subtracted from the readings. The cardiolipin concentration was calculated for each sample using the equation C = B/V × D where B is the amount of cardiolipin in the sample well from the standard curve, V is the volume of sample added into the well, and D is the dilution factor. Since our samples were not diluted, the equation used was simply C = B/V. The concentration values were graphed in Prism 6.07 and were analyzed via one-way ANOVA at each timepoint.

## 4. Discussion

The mitochondrial dysfunction pathway was prominent in the initial IPA analysis of the liver transcriptomic datasets from the HZE-irradiated animals; further analysis identified several other prominent pathways which were directly linked to mitochondrial function, i.e., sirtuin signaling, oxidative phosphorylation, FXR/RXR activation, unfolded protein response, and ER stress. Many of these pathways were identified in the top five transcript canonical pathways in the majority of the HZE-irradiated transcriptomic datasets (Table 2). The proteomic datasets also picked up on many of the same pathways that were crucial to mitochondrial function, i.e., sirtuin signaling and LXR/RXR activation, but mitochondrial dysfunction was not in the top five proteomic canonical pathways. At first this was discerning, therefore, we focused on proteins that we identified in the proteomic data that specifically were involved in the mitochondrial dysfunction pathway (Table 1). This direct approach identified several proteins in several of the irradiated timepoints which supported the transcriptomic mitochondrial dysfunction data, but not all timepoints and treatments. In some treatments/timepoints, we identified no proteins involved in that pathway. In retrospect, this is not surprising because our proteomic analysis was performed on whole cell extracts. The transcriptomic analysis identified the mitochondrial dysfunction pathway because many mitochondrial RNAs are transcribed in the nucleus, thus, the deep RNA sequencing picked up on them. The mitochondrial proteins are in the organelle and many of them get diluted in the whole cell protein extraction, only the most abundant mitochondrial proteins are identified in whole cell proteomic analysis. If the proteomic analysis had been performed on isolated mitochondria, the proteomic results would have been more mitochondrial centric.

The proteomic data identified activation of the immunological pathways that are among the top five canonical proteomic pathways after HZE irradiation, i.e., acute phase response signaling and JAK family kinase IL-6 type cytokine signaling pathways. This supports findings from previous work that used unbiased computational mathematical analysis of early transcriptomic data from ^56^Fe-irradiated mouse livers and showed activation of both immunological pathways and mitochondrial dysfunction pathways post-irradiation [22].

In the data analysis, it is important to focus on the top five canonical pathways identified, and also to note the other interesting, dysregulated transcripts/proteins and pathways listed in Table 2. The pathways identified by the transcriptomic and proteomic data are complementary and round out and support the mitochondrial dysfunction induced by HZE exposure and give insight into some possible countermeasure therapeutic targets for HZE exposure, some of which will be discussed below. The lipidomic data also support the mitochondrial dysfunction induced by HZE, and the Complex I assay shows significant and prolonged inhibition of this crucial enzyme in oxidative phosphorylation post HZE irradiation.

Within sirtuin signaling, there are seven sirtuins found in mammals that are involved in distinct metabolic and stress response pathways. Three of these sirtuins (SIRT3, -4, and -5) are localized in the mitochondria. These sirtuins are known to participate in the regulation of ATP production, metabolism, apoptosis, and cell signaling [23]. While the genes encoding for these specific sirtuins were not dysregulated in the transcriptomic data, two sirtuins (SIRT3 and -5) were identified in the proteomic data. The sirtuin signaling pathway is a large complex that is tightly linked to mitochondrial function and is involved in many processes including cell proliferation, tumor growth, glycolysis, cholesterol efflux, inflammation, ROS production, autophagy, oxidative stress, apoptosis, fatty acid oxidation, liver gluconeogenesis, and other responses that have been associated with radiation exposure. The NAD+ dependence of sirtuins has led to the belief that they are metabolic sensors due to their high levels observed when NAD+ is in abundance, as seen in times of nutrient stress. Hepatic SIRT3 levels have been found to be increased during times of fasting, and SIRT3 activates hepatic lipid catabolism. *Sirt3^−/−^* mutant studies have shown decreased fatty acid oxidation, low ATP production, and the animals have developed fatty liver and shown defects in thermogenesis and hypoglycemia during cold tests. SIRT3 is intimately involved in deacetylation reactions and numerous TCA cycle enzymes are modified by acetylation. SIRT3 has been shown to interact with and deacetylate Complex I subunits and succinate dehydrogenase in Complex II within the oxidative phosphorylation cascade. SIRT3’s interactions with succinate dehydrogenase and isocitrate dehydrogenase 2 influence the TCA cycle indirectly via deacetylation and activation of AceCS2 and glutamate dehydrogenase. In previous proteomic studies, SIRT3 has been shown to bind ATP synthase and it regulates mitochondrial translation which affects electron transport. Changes in SIRT3 expression have been associated with ROS production and scavenging. There is also support for SIRT3 to be pro-apoptotic as well as a tumor suppressor. However, some studies have also found it to be anti-apoptotic [23]. In our proteomic studies, SIRT3 was found to be upregulated at 9 months post-^28^Si irradiation and at 12 month post-^56^Fe irradiation. It was downregulated at 2 months post-3 Gy gamma and -^16^O irradiation, at 9 months post-^6^O, -^28^Si, and -3 Gy gamma irradiation, and at 12 months post-1 Gy gamma irradiation.

SIRT5 is known to physically interact with cytochrome C, but the significance of this interaction is still unknown. SIRT5 regulates carbamoyl phosphate synthetase which is the rate-limiting and first step in the urea cycle. Thus, SIRT5 coordinates with the detoxification of hepatic by-products of amino acid catabolism [23]. SIRT5 was upregulated at 1 month post-^16^O irradiation, at 9 months post-^56^Fe irradiation, and at 12 months post-^28^Si irradiation. It was downregulated at 9 months post-^16^O, -^28^Si, and -1 Gy gamma irradiation.

The ER is responsible for the secretion and synthesis of membrane proteins. Once the proteins are properly folded, then, they are passed on to the Golgi apparatus. Unfolded or misfolded proteins, however, are retained in the ER where they are degraded. If these unfolded proteins build up, the expression of ER chaperons and components of the machinery to degrade unfolded proteins are upregulated. This process is referred to as the ER stress response [24]. Organelle crosstalk is very important in relation to tumorigenesis as they constitute a complicated network with one another. Thus, the dysregulation of one of the downstream pathways may lead to severe mitochondrial dysfunction that would ultimately result in failure to properly regulate energy metabolism as well as ion buffering [25]. As mitochondria are able to synthesize some of their own proteins, they also have an autoregulatory mechanism, which is similar to the ER stress response, that is induced when there is an accumulation of unfolded proteins, known as the unfolded protein response [24]. Mitochondrial proteostasis is also regulated by other stress responsive signaling mechanisms. When eukaryotic initiation factor 2 (EIF2α) is phosphorylated, it induces attenuation of protein synthesis and activates other stress-responsive transcription factors [26]. The activation of the EIF2 signaling pathways were found in the top five canonical pathways of the early post-irradiation ^18^O (2, 6 and 9 months), ^56^Fe (2 months) and 1 and 3 Gy gamma (2 months) samples in the transcriptomic data. The EIF2 signaling pathway was identified in the ^56^Fe (4 months) sample in the transcriptomic data but was not in the top five canonical pathways.

Excessive ROS accumulation is a well-known response after HZE exposure. Nuclear factor erythroid (Nrf2) helps govern the expression of endogenous antioxidant synthesis and ROS-eliminating enzymes. Accumulating evidence shows that mitochondrial ROS activates Nrf2 which ultimately induces the expression of antioxidant genes as well as genes that are involved in mitochondrial quality and quantity control [7]. The NrF2-mediated oxidative stress response pathway was identified in several of the early timepoint HZE treatments, but was only in the top five canonical pathways in the ^56^Fe (4 and 9 months) and the 3 Gy gamma (2 months) samples in the transcriptomic data.

The L-carnitine shuttle is critical because the inner mitochondrial membrane is impermeable to fatty acyl-CoA thioesters. Thus, for fatty acids to be transported across the inner mitochondrial membrane, the carnitine shuttle is required [27]. The mitochondrial matrix is the site of the TCA cycle, and as previously mentioned, mitochondria also have a central role in amino acid metabolism via deamination and transamination [28]. The transcripts for carnitine palmitoyltransferase 1A were dysregulated in the post-irradiated ^18^O (1 and 2 months), ^56^Fe (2 months), and 3 Gy gamma (2 months) samples in the mitochondrial dysfunction pathway. The carnitine shuttle pathway was dysregulated in the post-irradiated ^56^Fe (1 month) transcriptomic data and in the ^18^O (12 months) and ^56^Fe (4 months) proteomic data.

Farnesoid X receptor (FXR) plays an important role in the maintenance of energy homeostasis as well as the integrity of organs such as the liver. It helps regulate bile acid, lipid, and glucose metabolism. Liver cancers were spontaneously developed in mice in the absence of FXR [29]. Liver X receptors (LXRs) are nuclear receptors that are involved in transcriptional control of lipid metabolism as well as function as nuclear cholesterol sensors that are activated in response to elevated intracellular cholesterol levels. They have been found to modulate immune and inflammatory responses in macrophages [30]. The FXR/RXR and LXR/RXR pathways are activated in both the transcriptomic and proteomic datasets in many of the irradiated datasets. This is most likely a compensatory mechanism to maintain energy homeostasis in the presence of mitochondrial dysfunction.

Coenzyme Q10 (CoQ_10_) is an essential electron transporter in Complexes I, II, and III. Ubiquinone-10 is its oxidized state, and it is enzymatically reduced to ubiquinol-10 which acts as the primary fat-soluble antioxidant that effectively protects membrane lipids, lipoproteins, and nucleic acids from oxidative damage. Thus, scavenging of ROS is essential for optimal mitochondrial function. Our transcriptomic data within the mitochondrial dysfunction pathway showed increased gene activation of ubiquinol-cytochrome c reductase and/or NADH as follows: ubiquinone oxidoreductase subunits in the post-irradiated ^18^O (at 1, 2, 4, and 9 months), ^56^Fe (at 2 months), 3 Gy gamma (at 2 and 9 months), and 1 Gy gamma (at 12 months) samples. Ubiquinome oxidative reductase protein was identified in the post-irradiated ^18^O (1 and 2 months), ^28^Si (9 and 12 months), and 1 Gy gamma (4 and 12 months) samples in the targeted proteins involved in the mitochondrial dysfunction pathway (Table 1). The ubiquinol-10 biosynthesis pathway was prevalent in the transcriptomic data in several of the HZE treatments and in the 1-, 2-, and 4-month post-irradiation with 1 Gy gamma. With normal aging, ubiquinol-10 levels and its biosynthesis have been observed to decrease. Thus, it is hypothesized that ubiquinol-10 may have anti-aging effects. Ubiquinol-10 is also believed to induce pathways that activate SIRT1, SIRT3, and peroxisome proliferator-activated receptor gamma coactivator 1α (Pparg), in addition to its influences on mitochondrial function [31].

It has been proposed that premature aging could potentially be an effect of HZE irradiation [32]. Mitochondria have been increasingly recognized as important players in the aging process and most aging-associated diseases have mitochondrial involvement [33]. Aging, in general, is known to result in biochemical and functional alterations within the mitochondrial electron transport chain resulting in reduced efficiency of electron transport as well as reduction in antioxidant activity, and an increase in oxidative stress [8]. In particular, the catalytic activity of Complexes I, III, and IV have all been observed to decline with age in liver as well as brain, heart, and skeletal muscle [11]. The Complex I data reported here infers relevance to the idea that HZE exposure may promote premature aging. At the one-month post-irradiation there is a large gap between Complex I function for ^56^Fe and ^16^O as compared with the sham control. However, at 9 months, this gap begins to lessen as the activity of Complex I begins to drop in the non-irradiated control mice.

A study conducted in yeast, identified 17 genes that are required for efficient uptake and/or transport of sterols. Sterols are synthesized in the ER and need to be efficiently transported to the plasma membrane which harbors ~90% of the free sterol pool of the cell. When sterols are taken up from the environment, they are transported from the plasma membrane to the ER where they are esterified to steryl esters. Of these 17 genes, many are required for mitochondrial function. Thus, it is thought there is a possible connection between mitochondrial biogenesis and sterol biosynthesis and uptake [34]. Sterol contents in organelle membranes are typically strictly controlled, and a fraction of excess sterols are esterified and stored as sterol esters in lipid droplets [10]. Because sterol esters are typically in low abundance, an increase in sterol ester within the mitochondria will result in mitochondrial dysfunction [35]. The sterol esters were elevated in the one-month post-irradiation ^56^Fe samples (Figure 2) which further supports mitochondrial dysfunction. Esterification of sterols will also interfere with cholesterol biosynthesis which is a pathway that was identified in the transcriptomic and proteomic data.

Phosphatidic acid (PA) is a second messenger lipid that has many signaling functions such as cell growth, proliferation, reproduction, and responses to hormones and stress [36]. PA also has a connection to the mitochondria as it inhibits mitochondrial division and stimulates mitochondrial outer membrane fusion. Mitochondrial fusion and division play important roles in mitochondrial size, number, distribution, function, and turnover [37]. PA is essential for controlling mitochondrial morphology as it is needed to form the curvature of the mitochondrial membrane that is necessary for mitochondrial interaction with the ER [38]. PA is also the precursor for the synthesis of cardiolipins [39]. Thus, if PA decreases so will the cardiolipins. The PA levels were reduced in the one-month post-irradiation ^56^Fe and ^16^O samples (Figure 2) which further supports mitochondrial dysfunction.

The mitochondria are in close interaction with the endoplasmic reticulum (ER). While their membranes are not directly fused (so they can retain their individuality), they do have contact points known as mitochondrial-associated ER membranes (MAMs) that make relatively stable connections between the organelles. This allows them to coordinate cellular functions such as calcium signaling, apoptosis, ER stress response, phospholipid synthesis, as well as translocation of phospholipid from the ER to mitochondrial membrane [25]. Most phospholipids such as PE, PS, and PC must be synthesized in the ER and must be imported into the mitochondria. PE can be produced within the mitochondria, but it requires the import of PS which must, then, be decarboxylated to PE [39]. Mitochondria and ER are also both important storage vessels of calcium and the transfer between them is crucial for both cell life and death. Calcium transfer between the organelles can be halted by simply increasing the distance of the MAMs. The decrease in PAs seen in the lipid data after HZE exposure (Figure 2) will disrupt the MAM contacts due to the PA’s effect on the mitochondrial membrane curvature, further supporting mitochondrial dysfunction.

CPA is a cyclic phosphatidic acid which has been shown to have specific biological functions such as antimitogenic regulation of cell cycle, regulation of actin stress fiber formation and rearrangement, inhibition of cancer cell invasion and metastasis, and mobilization of intercellular calcium [40]. CPA is also an antagonist at the peroxisome proliferator-activated receptor γ (PPARγ). The PPARγ binds to the retinoid X receptors (RXR), and then acts as a transcription factor to initiate cell proliferation and inflammation [41]. Lysophospholipids and leukotrienes are agonists at the PPARγ receptor [41]. Thus, an increase in CPA (Figure 2) infers a potential compensatory mechanism to shut down the effects of the proliferation and immune response from irradiation. Activation of the PPARγ and RXR pathways were also identified in the transcriptomic and proteomic data. LPI has been shown to be involved in cell growth, differentiation, motility and is known to be involved in metabolism and glucose homeostasis [42].

Lysophospholipids are the product of the activity of phospholipase A2 (PLA2) on phospholipids [42]. They are more hydrophilic and versatile than their corresponding phospholipids. These lipids can act as extracellular mediators by activating specific G-protein coupled receptors (GPCR) [43]. They have emerged as second-messenger molecules that can regulate intracellular signaling pathways that are involved in several physiological and pathological functions which include inflammation, angiogenesis, nervous system regulation, atherosclerosis, and tumorigenesis [42]. Accumulation of lysophospholipids can also have harmful effects on the structure and function of mitochondria, and high blood levels of lysophospholipids is a known indicator of mitochondrial dysfunction [35]. Several lysophospholipids were elevated after HZE irradiation (Figure 2) in our studies, with the highest levels observed in response to exposure to ^56^Fe.

Previous studies conducted in our lab at 6 months post ^56^Fe irradiation, showed an upregulation of the mouse analogue of GM2 in samples of irradiated livers. GM2 has been reported to be highly elevated (20–100 fold) in serum of human patients with hepatocellular carcinoma (HCC) [44]. In this study, the mouse analogue of human GM2 was upregulated in the HZE-irradiated samples and was highest in the ^56^Fe- and ^28^Si-irradiated samples (Figure 2). We propose that human GM2 may serve as a biomarker for early detection of HCC in astronauts during deep space missions.

The Complex I functional assay data, reported here, clearly support HZE-induced mitochondrial dysfunction, and thus supports the transcriptomic, proteomic, and lipidomic data. Beginning with the earliest timepoint, both ^16^O and ^56^Fe irradiation clearly reduced Complex I activity as compared with the sham control and maintained the reduction in activity throughout the time course.

The results presented here are just a fraction of the data that have been collected with a full systems biology interactive omics study. The power of such a study is that data are collected on multiple interactive pathways at several levels (transcripts, protein, lipids, and functional assays) and there are also specific data on tens of thousands of individual “players” (expressed genes, proteins/enzymes, and specific lipids) within the pathways. The data analyses are daunting but all these interacting parts help to identify specific therapeutic targets.

The primary pathway induced by HZE exposure is mitochondrial dysfunction. Many of the other prominent pathways identified are also involved in mitochondrial function and are probably activated as compensatory mechanism to support mitochondrial function. The ubiquinol-10 biosynthesis pathway is a primary example. The connection between ROS and HZE exposure is well known. These data explain that the primary sources of these ROS are from the dysfunctional mitochondria and the ubiquinol-10 biosynthesis pathway is trying to compensate by producing more ubiquinol-10 to scavenge more ROS to return to homeostasis. Many ROS scavengers are currently on the market as supplements. Other major pathways that are activated by HZE exposure are immunological pathways, many of which activate proinflammatory cytokines and/or lipids. On the basis of the data generated in this systems biology interactive omics study, the primary HZE countermeasure targets of interest include: ROS scavenger, mitochondrial centric, and anti-inflammatory.

### 4.1. ROS Scavengers

Ubiquinol-10 supplementation could be used as it has been shown to activate mitochondrial functions to decelerate senescence in senescence-accelerated mice. In that study, it was shown that ubiquinol-10 decreased the expression of sirtuin gene family members which resulted in the activation of peroxisome proliferator-activated receptor γ coactivator 1α that helps control mitochondrial biogenesis and respiration as well as the upregulation of superoxide dismutase 2 and isocitrate dehydrogenase 2 which are mitochondrial antioxidants. Supplementation with ubiquinol-10 was also found to increase activity in the mitochondrial complex I [31]. In addition to its major role in the electron transport chain, it also has an important antioxidant role which helps stabilize the plasma membranes as well as protect membrane phospholipids from peroxidation. Decreased levels of ubiquinol-10 in aging likely help contribute to membrane peroxidation injury. Chronic inflammation is also a common problem in relation to aging. By reducing the free radicals, it also helps reduce NF-κB which ultimately reduces the release of proinflammatory cytokines, in particular, tumor necrosis factor alpha (TNF-α) and interleukin-6 (IL-6) [8]. Since endogenous decreases in ubiquinol-10 are thought to be age related and it is inferred that HZE induces a premature aging component, supplementation with ubiquinol-10 may also be of great benefit to help protect against space irradiation.

N-acetylcysteine is an approved supplement that is the precursor of glutathione and is a potent antioxidant.

Pyrroloquinone (methoxatin) supplementation was approved by the FDA in 2008 as an antioxidant, it is touted to promote mitochondriogenesis and is proposed to stimulate nerve growth factor, and thus could have neuroprotective benefits [45].

Pterostibene (Resveratrol) supplementation is a proposed ROS inhibitor and increases mitochondrial function and biogenesis by activating SIRT1/AMPK/PGC pathways to counteract oxidative stress.

### 4.2. Mitochondrial Centric

Dimethyl fumarate is an FDA approved drug that is currently in use for the treatment of multiple sclerosis and is marketed under a variety of trade names. The mechanism of action is that it enhances mitochondrial biogenesis through the stimulation of the transcription factor NrF2 and it is anti-inflammatory and has cytoprotective properties [46]. Dimethyl fumarate has also been shown to protect mitochondria in cardiomyocytes from lipolysaccharide-induced damage [47], and thus may also help with HZE-induced cardiomyopathy.

One of the more promising mitochondrial function enhancing drugs is elamipretide (D-Arg-Dmt-Lys-Phe-NH_2_) [48]. Elamipretide is a D-amino acid tetrapeptide which prolongs a compound’s stability and function in a biological environment because D-amino acids are much less susceptible to enzymatic processing as compared with labile L-amino acids which are typically found in nature. Elamipretide is a novel mitochondria-targeted antioxidant peptide, which has protective effects against mitochondrial dysfunction and oxidative stress. Its dimethyltyrosine residue allows for scavenging of oxyradicals as well as inhibiting linoleic acid and low-density lipoprotein oxidation [48]. It ultimately eliminates ROS and increases ATP production by maintaining membrane potential. By reducing ROS, it can prevent the opening of the mitochondria permeability transition pore, prevent mitochondrial swelling, and reduce cytochrome c release in response to high Ca^2+^ overload. Elamipretide is known to selectively target the inner mitochondrial membrane by binding cardiolipins selectively via electrostatic and hydrophobic interactions. By interacting with cardiolipins, elamipretide prevents them from converting cytochrome c into a peroxidase, thus, protecting its electron carrying function, which in turn protects the structure of the mitochondrial cristae and promotes oxidative phosphorylation.

Unfortunately, elamipretide is not FDA approved, but it has been evaluated in humans and is well tolerated. Elamipretide enhances mitochondrial function, but cannot compensate for mitochondrial depletion. This does not discount the possibility of using this drug for a potential countermeasure or possibly even a radio protectant. It is also intriguing that this compound has previously been targeted to neurodegenerative disease and inflammatory disease, and thus this compound may be useful in combatting cognitive and inflammatory HZE-induced effects.

### 4.3. Anti-Inflammatory

Zileutin is an FDA approved 5-lipoxygenase (5-LO) inhibitor for asthma. By inhibiting 5-LO, zileutin blocks the formation of proinflammatory and tumor promoting leukotrienes and HETES [49]. The leukotrienes and HETES are derivatives of arachidonic acid (AA) which are released by phospholipase A2 (PLA2) [50]. PLA2 is also involved in the production of the lysophospholipids which were upregulated in the HZE-irradiated animals in this study. AA is metabolized to eicosanoids by three pathways, the COX pathway to prostaglandins, the P450 pathways to HETE/EETs, and the lipoxygenase pathways to the leukotrienes and HETEs. Targeting the COX pathway with aspirin is currently under investigation by NASA as a potential countermeasure for HZE-induced effects. Targeting the lipoxygenase pathway with zileuton will reduce inflammation induced by HZE exposure by reducing inflammatory leukotrienes. Leukotrienes also promote tumor production and differentiation, and thus zileuton is a proposed anticancer compound [50]. Finally, zileuton has been demonstrated to inhibit the phosphorylation of TAU protein which is necessary to initiate the aggregation of TAU protein which forms the neurofibrillary tangles in neurodegenerative diseases such as Alzheimer’s [51]. Thus, zileuton has the potential to block HZE-induced cognitive effects too.

## 5. Conclusions

Laiakis et al. [52] recently proposed HZE-induced mitochondrial dysfunction based on HZE-induced metabolite changes in mouse spleen. Mitochondrial stress was also recently proposed in a comprehensive multi-omics analysis from 59 astronauts and hundreds of samples that have been on space missions [53]. The space missions research was not HZE based, but was pivotal in illustrating the effects of being in a spacecraft in orbit for extended periods in which the inhabitants are exposed to extended microgravity, decreased partial pressure O_2_, increased CO_2_ concentration, and other flight stressors, i.e., tight quarters, sleep deprivation, and psychological stress, all of which influenced mitochondrial function, enhanced the immune response, and altered cell cycle events. The integrated omics study of HZE-induced microenvironmental changes in mouse, presented here, definitively demonstrates that mitochondrial dysfunction is induced by low dose HZE irradiation. Mitochondrial dysfunction is a major concern for the health and safety of deep space astronauts. Mitochondrial dysfunction in liver is not the only HZE-induced concern, these effects will also be very detrimental in brain and cardiac tissues which have high cellular concentrations of mitochondria.

As we look to travel deeper into our galaxy, mitochondrial effects are of great risk for these missions as the HZE contained within GCR effects could be additive with the effects that have been seen on the ISS from factors such as microgravity, dehydration, hypoxia, stress, and high levels of microbial containment [54,55,56]. One other factor that could exacerbate these problems further once on the surface of the moon, Mars, or other stellar body is exposure to space dust. Lunar hay fever was a term coined during the Apollo moon walks, which was an allergic reaction to lunar dust that the astronauts brought into the space craft on their suits after surface exploration. Other studies have shown that exposure to meteorite dusts induces increases in ROS and inflammatory responses [57] which is most likely linked to the high levels of iron (III) oxides in meteorite dusts [58]. The impact of GCR exposure, microgravity, space flight stressors, and exposure to immunogenic space dusts will minimally be additive to the deep space traveler. Countermeasures targeted at enhancing mitochondrial function, reducing ROS, and reducing unbridled immune response induced by GCRs, microgravity, in-flight stressors, and exposure to “novel space antigens” such as space dusts will be essential to reduce cognitive decline, cardiac toxicity, and carcinogenesis during and after deep space journeys.

## Figures and Tables

**Table 1:** Altered Transcripts and Proteins in the Mitochondria Dysfunction Pathway.

Conditions with Mitochondria Dysfunction Pathway Effected	Number of Transcripts in the Pathway	Dysregulated Transcripts within Pathway	Number of Proteins in the Pathway	Dysregulated Proteins within Pathway
1 mo ^18^O	46	aconitase 2, ATP synthase (H+ transporting mito F1 complex epsilon subunit, F1 subunit alpha beta & gamma, membrane subunit F, & peripheral stalk subunit OSCP), cytochrome c oxidase (subunit4I1, 6C, 7A2 like), carnitine palmitoyltransferase 1A, cytochrome c1, glutathione peroxidase 4, glutathione-disulfide reductase, cytochrome b, MTND2, NADH dehydrogenase subunits 4, 5 & 6 (complex I), NADH:ubiquinone oxidoreductase subunits A9, A10, A11, A13, B3, B4, B9, core subunit V1 & V2, pyruvate dehydrogenase E1 alpha 1 subunit, PTEN induced putative kinase 1, peroxiredoxin 3 & 5, succinate dehydrogenase complex iron sulfur subunit B, subunit C, & subunit D, superoxide dismutase 2, ubiquinol-cytochrome c reductase complex III subunit X, core protein 1, core protein 2 & Rieske iron-sulfur polypeptide 1, voltage dependent anion channel 1 & 2	8	ATP synthase membrane subunit f, catalase, NADH dehydrogenase subunit 1 (complex I), NADH:ubiquinone oxidoreductase subunit V3, peroxiredoxin 3, synuclein alpha, SURF1 cytochrome c oxidase assembly factor, xanthine dehydrogenase
1 mo ^56^Fe	9	ATP synthase F1 subunit gamma, carnitine palmitoyltransferase 1A, cytochrome b, NADH dehydrogenase subunit 1, 4, 5 & 6 (complex I), MTND2, NADH:ubiquinone oxidoreductase subunit B4	0	
1 mo ^28^Si	3	cytochrome b, MTND2, NADH dehydrogenase subunit 6 (complex I)	6	ATP synthase F1 subunit delta, catalase, mitogen-activated protein kinase 9, NADH dehydrogenase subunit 4(complex I), nicastrin, ras homolog family member T2
1 mo 3 Gy	0		7	ATP synthase membrane subunit f, catalase, NADH dehydrogenase subunit 1 (complex I), MTND2, NADH dehydrogenase subunit 4 (complex I), peroxiredoxin 3, synuclein alpha
2 mo ^18^O	37	ATP synthase (H+ transporting mito F1 complex epsilon subunit, F1 subunit alpha beta, gamma & delta, membrane subunit c locus 1 membrane subunit F & G, & peripheral stalk subunit OSCP & D), cytochrome c oxidase (subunit4I1, 6B1, 6C, 7B, & 8A), carnitine palmitoyltransferase 1A, cytochrome c1, glutathione peroxidase 4, glutathione-disulfide reductase, leucine rich repeat kinase 2, NADH dehydrogenase subunits 4 (complex I), NADH:ubiquinone oxidoreductase subunits A1, A2, A6, A8, A9, A10, A12, A13, B4, B7, B9, B11, S4, V3 core subunit S2 V1 & V2, oxoglutarate dehydrogenase, PTEN induced putative kinase 1, peroxiredoxin 3 & 5, parkin RBR E3 ubiquitin protein ligase, succinate dehydrogenase complex iron sulfur subunit B, subunit C, & subunit D, ubiquinol-cytochrome c reductase complex III subunit X, core protein 2 & Rieske iron-sulfur polypeptide 1, voltage dependent anion channel 1	10	ATP synthase membrane subunit f, catalase, cytochrome c oxidase subunit 7A2 like, mitogen-activated protein kinase, kinase 4, mitogen-activated protein kinase 9, NADH:ubiquinone oxidoreductase subunit A11, NADH:ubiquinone oxidoreductase complex assembly factor 1, NADH:ubiquinone oxidoreductase subunit B6, peroxiredoxin 3, thioredoxin reductase 2
2 mo ^56^Fe	19	aph-1 homolog B gamma-secretase subunit, ATP synthase F1 subunit gamma, ATP synthase membrane subunit g, ATP synthase peripheral stalk subunit OSCP, cytochrome c oxidase copper chaperone COX17, cytochrome c oxidase subunit 4I1, cytochrome c oxidase subunit 6C, glutathione peroxidase 4, leucine rich repeat kinase 2, cytochrome b, NADH dehydrogenase subunit 4 (complex I), NADH:ubiquinone oxidoreductase subunits A1, A2, B2, B4, B9, & core subunit V2, synuclein alpha & ubiquinol-cytochrome c reductase binding protein	0	
2 mo 3 Gy	26	ATP synthase (H+ transporting mito F1 complex epsilon subunit, F1 subunit gamma & delta, membrane subunit F & G, & peripheral stalk subunit OSCP), cytochrome c oxidase (subunit4I1, 6C, 7A2 like, & 8A), carnitine palmitoyltransferase 1A, cytochrome c1, glutathione peroxidase 4, glutathione-disulfide reductase, cytochrome b, NADH dehydrogenase subunits 1,4,5 &6 (complex I), MTND2, NADH:ubiquinone oxidoreductase subunits A2, A6, A9, A13, B2, B9, core subunit S2, S7 & V1, PTEN induced putative kinase 1, peroxiredoxin 5, parkin RBR E3 ubiquitin protein ligase, succinate dehydrogenase complex subunit C & D, ubiquinol-cytochrome c reductase complex III subunit X & XI & Rieske iron-sulfur polypeptide 1	0	
4 mo ^18^O	11	ATP synthase membrane subunit f, ATP synthase peripheral stalk subunit OSCP, cytochrome c oxidase subunit 5A & 6B1, NADH:ubiquinone oxidoreductase subunit A3, A7, A11, A12 & S6, thioredoxin 2, ubiquinol-cytochrome c reductase complex III subunit X	0	
4 mo 3 Gy	6	cytochrome c oxidase subunit 5A & I, cytochrome b, MTND2, NADH dehydrogenase subunit 4 & 5 (complex I)	0	
4 mo 1 Gy	0		6	caspase 8, NADH dehydrogenase subunit 4 (complex I), nicastrin, NADH:ubiquinone oxidoreductase subunit B6, NADH:ubiquinone oxidoreductase core subunit S3, thioredoxin reductase 2
9 mo ^18^O	12	ATP synthase membrane subunit c locus 2, cytochrome c oxidase copper chaperone COX11, cytochrome c oxidase subunit 5A, glutathione peroxidase 4, cytochrome b, MTND2, NADH dehydrogenase subunit 4 & 5 (complex I), NADH:ubiquinone oxidoreductase subunit A8, A11, & S6, thioredoxin 2	0	
9 mo 3 Gy	7	cytochrome c oxidase copper chaperon COX11, cytochrome c oxidase subunit 1, MTND2, NADH dehydrogenase subunit 4 & 6 (complex 1), NADH:ubiquinone oxidoreductase subunit B4, ubiquinol-cytochrome c reductase binding protein	0	
9 mo ^28^Si	0		7	caspase 3, cytochrome c oxidase subunit 7A2 like, mitogen-activated protein kinase 9, MTND2, NADH dehydrogenase subunit 4 (complex I), NADH:ubiquinone oxidoreductase core subunit S3, ras homolog family member T2
12 mo ^28^Si	0		6	ATP synthase F1 subunit delta, catalase, cytochrome c oxidase subunit 6A1, MTND2, NADH:ubiquinone oxidoreductase complex assembly factor 2, ras homolog family member T2
12 mo 1 Gy	9	cytochrome c oxidase subunit 7A2, 7A2 like, & 8A, fission mitochondrial 1, furin paired basic amino acid cleaving enzyme, NADH:ubiquinone oxidoreductase subunit B9, S6 & core subunit S2	11	ATP synthase F1 subunit delta, catalase, cytochrome c oxidase assembly homolog COX15, cytochrome c oxidase subunit 7A2 like, mitogen-activated protein kinase 9, MTND2, NADH:ubiquinone oxidoreductase subunit A9, NADH:ubiquinone oxidoreductase complex assembly factor 1, NADH:ubiquinone oxidoreductase subunit B6, ras homolog family member T2, SURF1 cytochrome c oxidase assembly factor

**Table 2:** Pathways Effected in Each Condition Based on Transcripts and Proteins.

Condition	Top 5 Transcript Based Canonical Pathways	Top Upstream Regulators Transcript Based	Other interesting Dysregulated Transcripts and Pathways	Top 5 Protein Based Canonical Pathway	Top Upstream Regulators Protein Based	Other Interesting Dysregulated Proteins and Pathways
1 mo ^18^O	Mitochondrial dysfunction Oxidative phosphorylation Sirtuin Signaling pathway FXR/RXR activation Fatty Acid beta oxidation I	PPARA Pirin ixic acid POR TO-901317 Mono-(2-ethylhexyl) phthalate	TCA Cycle II, Gluconeogenesis I (aldolase, fructose-bisphosphate C; enolase 1; malate dehydrogenase 1 & 2, phosphoglycerate kinase 1 all down), ubiquinol-10 biosynthesis, mitochondrial L-carnitine shuttle pathway (acyl-coA synthetase long chain family member 1, carnitine palmitoyltransfease 1A & solute carrier family 27 member 5 all down), ceramide signaling	Sirtuin Signaling Pathway RhoA Signaling (cytoskeleton organization) Clathrin-mediated Endocytosis signaling LXR/RXR activation Actin Cytoskeleton Signaling	HNF4A-hepatocyte nuclear factor 4 alpha Insulin D-glucose Methylprednisolone ABCB6	PI3K (down) mTOR (up) cyclin dependent kinase 2(up) cyclin dependent kinase inhibitor 1B (down) glycogen synthase kinase 3 beta (down) insulin signaling receptor (down) mannose-6-phosphate receptor (down)
2 mo ^18^O	EIF2 signaling Oxidative phosphorylation Regulation of eIF4 and p70S6K signaling Mitochondrial dysfunction Sirtuin signaling pathway	MLXIPL RICTOR YAP1 MYC MYCN	mTOR signaling, Protein Ubiquitination Pathway, NRF2-mediated oxidative stress response, Unfolded protein response, TCA cycle II (fumarate hydratase, succinate dehydrogenase complex (iron sulfur subunit B, C & D all down)	Huntington’s Disease Signaling (HIP1 down) (HSP40 up) Iron homeostasis signaling pathway ILK signaling Tight Junction Signaling Glycogen Degradation III	HNF4A CST5 Maslinic acid miR-30c-5p (and other miRNAs w/seed GUAAACA) desmopressin	Also see sirtuin signaling and ceramide signaling (SMPD (sphingomyelin phosphodiesterase 4) up)
4 mo ^18^O	EIF2 signaling Unfolded protein response Regulation of eIF4 and p70S6K signaling Protein Ubiquitination Pathway ER stress pathway-CALR (calreticulin) down, DDIT3 (DNA damage inducible transcript 3) down, EIF2AK3 down, HSP90B1 down, HSPA5 down	XBP1 ERN1 Tunicamycin RICTOR ATF6	mTOR signaling, sirtuin signaling pathway	Acute phase response signaling Clathrin-mediated endocytosis signaling Phagosome maturation Role of JAK family kinases in IL-6 type cytokine signaling FXR/RXR activation	HNF4A CST5 TP53 Methylprednisolone D-glucose	Also see B cell receptor signaling, production of nitric oxide & ROS macrophages, cellular senescence (predicted inhibition), CDKN1B cyclin dependent kinase inhibitor (down), CDK2 cyclin dependent kinase 2-activation of s-phase progression, sumoylation pathway-SAE1(SUMO1 activating enzyme subunit 1), LXR/RXR activation, insulin receptor signaling
9 mo ^18^O	EIF2 signaling Mitochondrial dysfunction Oxidative phosphorylation Regulation of eIF4 and p70S6K signaling Sirtuin signaling pathway	MLXIPL RICTOR MYC YAP1 CTNNB1	mTOR signaling	Clathrin-mediated endocytosis signaling RhoA signaling Acute phase response signaling Huntington’s disease signaling Signaling by Rho family GTPases	NHF4A RHOJ XBP1 Bvr Sirolimus	Coenzyme A biosynthesis, Sirtuin signaling, heme oxygenase 1 & 2 (down)
12 mo ^18^O	Mouse Embryonic Stem Cell Pluripotency-ID1 (inhibitor of DNA binding), ID2 down Trans, trans-farnesyl diphosphate biosynthesis-FDPS (farnesyl disphosphate synthase) up Pregnenolone biosynthesis-CYP26A1 up Histidine Degradation VI-same Superpathway of Geranylgeranyldiphosphate biosynthesis I (via Mevalonate)-FDPS up	ACVR2A LTBP4 SMAD1/5 Notch BMP10	Ubiquinol-10 biosynthesis- CYP26A1 up, Role of lipids/lipid rafts in the pathogenesis of influenza-FDPS up	Acute phase response signaling Tight junction signaling IL-8 signaling LXR/RXR activation L-glutamine Biosynthesis II(tRNA-dependent)	HNF4A CST5 Tetrachlorodibenzodioxin GPD1 SLC25A13	L-carnitine shuttle pathway (acyl-CoA synthetase long chain family member 3 (down)) & member 4 (up), phospholipase c signaling, type II diabetes signaling, role of NFAT in regulation of the immune response, CDK2 (down), Unfolded protein response
1 mo ^56^Fe	PXR/RXR activation LPS/IL-1 mediated inhibition of RXR Function Sirtuin Signaling Pathway Nicotine Degradation II Circadian Rhythm Signaling-ARNTL (aryl hydrocarbon receptor nuclear translocator) up, BHLHE40 (basic helix-loop-helix family member e40) down; CLOCK (clock circadian regulator) up; CRY1 (cryptochrome circadian regulator 1) up; PER1 (period circadian regulator 1) down; PER2 down; PER3 down	RORC RORA PPARA Methylprednisolone NR1I2	Ubiquinol-10 biosynthesis, acyl-CoA hydrolysis	Acute phase response signaling Glycogen degradation III Phagosome maturation Huntington’s Disease signaling Clathrin-mediated endocytosis signaling	TO-901317 Ciprofibrate Nitrofurantoin SCAP ACOX1	Calcium transport I (ATPase sarcoplasmic/ER Ca2+ transporting 2 (down) & ATPase plasma membrane Ca2+ transporting 1 (up)), LXR/RXR (apolipoprotein A5-activates cholesterol efflux), Death receptor signaling (CASP7 inhibits DNA repair (down))
2 mo ^56^Fe	EIF2 Signaling Acute phase response signaling-exported Regulation of eIF4 and p70S6K signaling-exported list mTOR signaling-exported Production of nitric oxide and ROS in macrophages	Lipopolysaccharide MLXIPL IL1B YAP1 IFNG	Sirtuin signaling pathway, FXR/RXR, NRF2-mediated oxidative stress response	Acute phase response signaling Xenobiotic metabolism signaling NAD salvage pathway II UVB-induced MAPK signaling NF-kB activation by viruses	HNF4A Pregnenolone carbonitrile Pirinixic acid Estrone RAB1B	ceramide signaling (sphingosine kinase 2 (up))
4 mo ^56^Fe	Unfolded protein response Aldosterone signaling in Epithelial cells Endoplasmic reticulum stress pathway Protein ubiquitination pathway NRF2-mediated oxidative stress response	XBP1 Tunicamycin ERN1 ATF6 1,2-dithiol-3-thione	EIF2 signaling, Regulation of eIF4 and p70S6K signaling mTOR, Glucocorticoid receptor signaling, Sirtuin signaling pathway, insulin receptor signaling	Cell cycle regulation by BTG family proteins Inhibition of ARE-mediated mRNA degradation pathway 5-aminoimidazole ribonucleotide biosynthesis I Dopamine receptor signaling Breast cancer regulation by Stathmin1	HNF4A Camptothecin (+-)-2-hydroxyoleic acid FAS HNRNPH1	Cyclins and cell cycle regulation, regulation of eIF4 & p70S6K signaling, ceramide signaling (different protein that O) sphingosine kinase 2, role of CHK proteins in cell cycle checkpoint, mitochondrial L-carnitine shuttle pathway (acyl-CoA synthetase long chain family member 4 down & solute carrier family 27 member 4 (down), thrombin signaling, mTOR signaling
9 mo ^56^Fe	Unfolded protein response-CEBPE down, all up ERN1, HSPA8, Hspa1b, HSPH1, MAP3K5 NRF2-mediated oxidative stress response-ACTG1, BACH1, DNAJA1, DNAJB1, FKBP5, GSTP1, MAP3K5 all up Aldosterone signaling in epithelial cells-DNAJA1, DNAJB1, HSP90AA1, HSPA8, HSPA4L, HSPH1 all up Protein Ubiquitination pathway same as above Agranulocyte-adhesion and diapedesis-ACTG1 up, CLDN3 down, CXCL2 (C-X-C chemokine ligand) up, CXCL13 up, IL1R1 (interleukin 1 receptor type 1) up	NR5A2 Lipopolysaccharide Cisplatin Thapsigargin NR1I3	GADD45 signaling-CDKN1A (cyclin dependent kinase inhibitor 1A) up, GADD45G (growth arrest and DNA damage inducible gamma) up LXR/RXR signaling-C4A/C4B (complement C4B), CYP7A1, HMGCR (3-hydroxy-3-methylgutaryl-CoA reductase), IL1R1 (interleukin 1 receptor type 1) all up Asparagine biosynthesis I-ASNS(asparagine synthetase glutamine-hydrolyzing) up, FXR/RXR-C4A/C4B, CYP7A1, FOXA3, SLC10A2 all up, PXR/RXR ER stress pathway-ALAS1, CYP7A1, IGFBP1 all up, PI3K/AKT signaling-CDKN1A up, GDF15 (growth differentiation factor 15) down, HSP90AA1 up, MAP3K5 up, Sirtuin signaling pathway-BCL2L11, GADD45G, MT-ND2, MT-ND4, MT-ND6, PFKFB3 (6-phosphofructo-2 kinase/fructose 2,6-biphosphatase 3) all up Apoptosis signaling-BCL2L11 up, ENDOG down, MAP3K5 up	Purine nucleotides de novo biosynthesis II 5-aminoimidazole ribonucleotide biosynthesis I Regulation of cellular mechanics by calpain protease Acute phase responses signaling Sertoli cell-Sertoli cell junction signaling	TO-901317 Ciprofibrate Diethyl nitrosamine HNF4A ACOX1	
12 mo ^56^Fe	only 6 genes dysregulated all up		Gm23442 Gm25394 Gm25835 Gm26397 Snora78 Snord13 (small nucleolar RNA)	Acute phase response signaling Mechanisms of viral exit from host cells Wnt/beta catenin signaling Endothelin-1 signaling Endometrial cancer signaling	HNF4A Let-7a-5p (and other miRNAs w/seed GAGGUAG) 1,2-dithiol-3-thione Methylprednisolone miR-30c-5p (other miRNAs w/seed GUAAACA)	Sirtuin signaling pathway
1 mo ^28^Si	Mitochondrial Dysfunction Asparagine Biosynthesis I-ASNS (asparagine synthetase (glutamine-hydrolyzing) down Sirtuin Signaling Pathway-MT-CYB; MT-ND2; MT-ND6Alpha tocopherol degradation-CYP4A11 Oxidative phosphorylation	Actinonin MRPL12 DAP3 MT-TM SIRT3	Ubiquinol-10 biosynthesis	Huntington’s Disease signaling Stearate Biosynthesis I (animals) FAT10 signaling pathway D-myo-inositol (1,4,5,6)-tetraphosphate biosynthesis D-myo-inositol (3,4,5,6)-tetrabiphosphate biosynthesis	HNF4A Glucagon Desmopressin CALCA BCAN	Acyl-CoA hydrolysis
2 mo ^28^Si	FXR/RXR activation-FOXA2 up, HPX down, RARA down, SAA1 * down Role of Oct4 in Mammalian Embryonic stem cell pluripotency-FOXA2 up, RARA down Acute phase response signaling-HP, HPX (hemopexin), SAA1 * all down TR/RXR activation-HP (haptoglobin) and THRSP (thyroid hormone responsive)	Lipopolysaccharide TNF RORC IL1B NR1H4		Acute phase response signaling Fcy receptor mediated phagocytosis in macrophages and monocytes Stearate biosynthesis I (animals) Caveolae-mediated endocytosis signaling Leukocyte extravasation signaling	HNF4A miR-1-3p(and other miRNAs w/seed GGAAUGU) ACOX1 ESR1 Fulvestrant	
4 mo ^28^Si	Unfolded protein response-DNAJB9, HSPA5, Hspa1b, SYVN1 (synoviolin 1) all down IL-7 signaling pathway-IGHG1, Ighg2b, Ighg2c all up Phagosome formation-IGHG1, Ighg2b, MARCO (macrophage receptor with collagenous structure) all up Autoimmune thyroid disease signaling-IGHG1, Ighg2b both up Hematopoiesis from pluripotent stem cells-same as above	CLOCK ATF6 Thapsigargin HTT tunicamycin	Phosphatidylethanolamine biosynthesis II (choline kinase alpha up), ER stress pathway (heat shock protein A(HSP70) member 5 down)	Huntington’s Disease Signaling 3-phoshoinsositide degradation D-myo-inositol-5-phosphate metabolism Superpathway of inositol phosphate compounds 3-phosphoinositide biosynthesis	HNF4A miR-30c-5p(and other miRNAs w/seed GUAAACA) PPARA KLF3 SREBF1	
9 mo ^28^Si	Acetone Degradation I (to Methylglyoxal)-CYP2A6 down, CYP2C8 down, CYP4A22 up Stearate biosynthesis I (animals)-ACOT1 down, CYP4A22 up, ELOVL6(ELOVL fatty acid elongase 6) down Nicotine Degradation III-CYP2A6, CYP2C8, UGT1A4 all down Melatonin Degradation I-same as above Nicotine Degradation II-same	STAT5B Pirinixic acid RORC L-triiodothyronine PPARA	Acyl-CoA hydrolysis, ubiquinol-10 biosynthesis	Remodeling of epithelial adherens junctions Caveolar-mediated endocytosis signaling Clathrin-mediated endocytosis signaling Paxillin signaling Integrin signaling	HNF4A ERBB3 IL4 RHOJ ciprofibrate	
12 mo ^28^Si	only 1 gene upregulated		Gm22154	Aldosterone signaling in epithelial cells Protein ubiquitination pathway Stearate Biosynthesis I (animals) Acute phase response signaling FAT10 signaling pathway	Pirinixic acid ELL2 HNF4A CYP2E1 ciprofibrate	sirtuin signaling pathway
1 mo 1 Gy	Stearate Biosynthesis I (animals)-ACOT1 (acyl-CoA thioesterase 1) down; ACOT4 down; CYP4A11 down; FASN (fatty acid synthase) up Superpathway of cholesterol biosynthesis-FDPS (farnesyl diphosphate synthase) up; MVD (mevalonate diphosphate decarboxylase) up; SQLE (squalene epoxidase) up Acyl-CoA hydrolysis-ACOT1; ACOT4 Superpathway of geranylgeranyl diphosphate biosynthesis I (via Mevalonate) TR/RXR activation-BCL3 (B cell CLL/lymphoma 3) up; FASN up; HP (haptoglobin) up	FGR19 PPARA SCAP CFTR SREBF1	Palmitate biosynthesis I, ubiquinol-10 biosynthesis	Sirtuin signaling pathway Antigen presentation pathway Phagosome maturation Remodeling of epithelia adherens junctions Molybdenum cofactor biosynthesis	HNF4A POR SREBF1 SCAP ESR1	PTEN signaling, sumoylation, death receptor signaling
2 mo 1 Gy	EIF2 signaling mTOR signaling Regulation of eIF4 and p70S6K signaling Unfolded protein response Complement system	MLXIPL Lipopolysaccharide YAP1 IL6 CTNNB1	Ubiquinol-10 biosynthesis, sphingosine-1-phosphate signaling	Stearate Biosynthesis I (animals) 3-phosphoinositide degradation Clathrin-mediated endocytosis signaling 3-phosphoinositide biosynthesis D-myo-inositol (1,4,5,6)-tetra bisphosphate biosynthesis	CFTR TO-901317 Pirinixic acid HNF4A PPARA	
4 mo 1 Gy	Unfolded protein response ER stress pathway-CALR, DDIT3, DNAJC3, EIF2AK3, HSP90B1, HSPA5, XBP1 all down LXR/RXR activation FXR/RXR activation Acute phase response signaling	XBP1 Tunicamycin ERN1 RORC RORA	NRF2-mediated oxidative stress response, acyl-coA hydrolysis, ubiquinol-10 biosynthesis, palmitate biosynthesis I (fatty acid synthase up), fatty acid biosynthesis Initiation II(same from palmitate pathway)	NAD Salvage Pathway II Cholesterol Biosynthesis I Cholesterol Biosynthesis II (via 24,25-dihydrolanosterol) Cholesterol Biosynthesis III (via Desmosterol) TWEAK signaling	HNF4A Methylprednisolone miR-155-5p (miRNAs w/seed UAAUGCU) PML DMD	
9 mo 1 Gy	Role of JAK2 in hormone-like cytokine signaling-IRS2 (insulin receptor substrate 2), SOCS2 (suppressor of cytokine signaling), SOCS3 all up Acute phase response signaling-IL1R1, SAA1 * (serum amyloid A1), SOCS2, SOCS3, all up TF (transferrin) down Sirtuin signaling pathway-ARNTL, CDH1, GADD45G, MT-ND4, MT-ND5 all up, MYCN (MYCN proto-oncogene, bHLH TF) down IGF-1 signaling-IGFBP1, IRS2, SOCS2, SOC3 all up IL-9 signaling-IRS2, SOCS2, SOCS3 all up	RORC Dexamethasone IL1B RORA tretinoin	PXR/RXR signaling-CYP2A6 down, CYP2B6 down, IGFBP1 up, LXR/RXR-IL1R1 up, SAA1 * up, TF down, FXR/RXR-SAA1 * up, SLC22A7 up, TF down Phosphatidylethanolamine biosynthesis II (choline kinase alpha up)	Glycine Betaine Degradation Clathrin-mediated endocytosis signaling Methionine salvage II (mammalian) L-serine degradation Caveolar-mediated endocytosis signaling	Diethylnitrosamine miR-155-3p (miRNAs w/seed UCCUACC) RORA TP53 NCOA6	Sirtuin signaling pathway, superoxide radicals degradation
12 mo 1 Gy	Mitochondrial dysfunction VDR/RXR activation-EP300, FOXO1, GADD45A, NCOR2, and PPARD all down, HR up B cell receptor signaling-9 molecules exported Estrogen receptor signaling-EP300, HNRNPD, NCOR2, RRAS, SPEN, TAF15, TAF4B all down Acute phase response signaling-IL1R1, IL6R, MAP3K5, RRAS, SAA1 *, SAA2-SAA4, SERPINA3, SOCS3 all down	ST1926 IL3 EPOR Lipopolysaccharide IL1B	Sirtuin signaling, mTOR	Stearate Biosynthesis I (animals) Acyl-CoA hydrolysis Acetone Degradation I (to methylglyoxal) Clathrin-mediated endocytosis signaling Sirtuin signaling pathway	NKX2-2-AS1 HNF4A Pirinixic acid Ciprofibrate di(2-ethylhexyl) phthalate	mitochondrial L-carnitine shuttle pathway
1 mo 3 Gy	IL-17A signaling in fibroblasts-CEBPB (CCAAT enhancer binding protein beta) up; CEBPD (delta) up; JUN (Jun proto-oncogene) up; LCN2 (lipocalin 2) down Glucocorticoid receptor signaling-AR (androgen receptor) up; CDKN1A (cyclin dependent kinase inhibitor 1A) up; CEBPB up; DUSP1 (dual specificity phosphatase 1) up; FKBP5 (FK506 binding protein 5) up; JUN up; TSC22D3 (TSC22 domain family member 3) up Acute phase response signaling-APCS (amyloid P component, serum) down; CEBPB up; IL6R (interleukin 6 receptor) up; JUN up; SAA1 (serum amyloid A1) down JAK/Stat Signaling-CDKN1A (cyclin dependent kinase inhibitor 1A) up; CEBPB up; Jun up Hepatic Fibrosis/Hepatic Stellate Cell Activation-COL15A1 (collagen type XV alpha 1 chain) down; COL25A1 (collagen type XXV alpha chain) down; CTGF (connective tissue growth factor) up, IL6R up	TNF IL1B IFNG Glucocorticoid dexamethasone	Sumoylation pathway	Remodeling of epithelial adherns junctions Clathrin-mediated endocytosis signaling Systemic lupus erythematosus signaling Fcy receptor-mediated phagocytosis in macrophages and monocytes Caveolar-mediated endocytosis signaling	HNF4A SREBF1 TLE3 miR-30c-5p (and other miRNAs w/seed GUAAACA) COMMD1	Insulin receptor signaling, sirtuin signaling pathway, mTOR signaling, PPAR signaling, PI3K/AKT signaling, ceramide signaling (sphingomyelin phosphodiesterase 4 (up)), PTEN Signaling
2 mo 3 Gy	EIF2 signaling Regulation of eIF4 and p70S6K signaling mTOR signaling Unfolded protein response NRF2-mediated oxidative stress response	MLXIPL YAP1 Tunicamycin XBP1 MYCN	Sirtuin signaling pathway, ceramide signaling, ER stress pathway	Acute phase response signaling Clathrin-mediated endocytosis signaling tRNA charging RhoGDI signaling Death Receptor signaling	Monobutyl phthalate HNF4A CST5 OGA 2,4,5,2′,4′,5′-heachlorobiphenyl	
4 mo 3 Gy	Oxidative phosphorylation Mito dysfunction Sirtuin signaling pathway-FOXO1, MT-CYB, MT-ND2, MT-ND4, MT-ND5, PPARGC1A all up, RRP9 down Unfolded protein response-HSPA5, Hspa1b, SYVN1 all down Granzyme B signaling-ENDOG (endonuclease G), LMNB2 (lamin B2) both down PXR/RXR activation-CYP2C8, FOXO1, PPARGC1A all up FXR/RXR activation-FOXO1 up, PPARGC1A up, SAA1 * down	IL1B THBS4 STAT3 ALKBH1 NSUN3		JAK/Stat signaling IL-7 signaling pathway Reelin Signaling in Neurons Clathrin-mediated endocytosis signaling Systemic lupus erythematosus signaling	HNF4A Glucagon SREBF1 Insulin PPARGC1B	Sirtuin signaling, sphigosine-1-phosphate signaling, ubiquinol-10 biosynthesis (coenzyme Q3, methyltransferase up & cytochrome P450 family 46 subfamily A member 1 (down))
9 mo 3 Gy	Mito dysfunction Oxidative phosphorylation IL-7 signaling pathway-IGHG1, Ighg2b, Ighg2c, MYC all up, JUN down Sirtuin signaling pathway-HIST1H4J & JUN down, MT-ND2, MT-ND4, MT-ND6, MYC, NDUFB4 all up LPS/IL-1 mediated inhibition of RXR function-ALAS1, CYP2A6, CYP7A1, GSTA5, Sult1d1 all up and JUN down	Lipopolysaccharide RORC NR1I2 RORA Cadmium chloride		Actin cytoskeleton signaling Acute phase response signaling Integrin signaling Signaling by Rho Family GTPases Remodeling of Epithelial Adherens Junctions	Desmopressin HNF4A Levodopa PPARA Gcg	Sirtuin signaling pathway, sphingosine-1-phosphate signaling
12 mo 3 Gy	Aryl hydrocarbon receptor signaling-DCT (dopachrome tautomerase) up, EP300, NCROR2, NFIA, NFIC, TP53 all down RAR activation-CYP26A1 up, ARID1A, EP300, NCOR2, ZBTB16 all down Iron homeostasis signaling pathway-ATP6V0C, EPAS1, IL6R all down, TFRC up Superpathway of cholesterol biosynthesis-DHCR7 down, FDPS up Hereditary breast cancer signaling-ARID1A, EP300, TP53 all down, UBC (ubiquitin) up	Diethylnitrosamine ACOX1 Hydrogen peroxide Pirinixic acid TAS-103	PPARalpha/RXRalpha-BCL3, EP300, NCOR2 all down, Cyp2c54 up, sumoylation pathway	Tight Junction Signaling RhoGDI Signaling Huntington’s Disease Signaling Mechanisms of Viral Exit from Host cells LXR/RXR activation	HNF4A CST5 SREBF1 D-glucose IPMK	

## Data Availability

The transcriptomic data used in this publication has been deposited in NCBI’s Gene Expression Omnibus (Nia et al., 2020) and are accessible through GEO Series accession number GSE136165 (https://www.ncbi.nlm.nih.gov/geo/query/acc.cgi?acc=GSE136165), (accessed on 29 October 2021).
